# Plasma Triggered Grain Coalescence for Self-Assembly of 3D Nanostructures

**DOI:** 10.1007/s40820-017-0130-z

**Published:** 2017-02-14

**Authors:** Chunhui Dai, Daeha Joung, Jeong-Hyun Cho

**Affiliations:** Department of Electrical and Computer Engineering, University of Minnesota, Minneapolis, MN 55455 USA

**Keywords:** 3D nanostructures, Grain coalescence, Etching profile, Self-assembly

## Abstract

Grain coalescence has been applied in many areas of nanofabrication technology, including modification of thin-film properties, nanowelding, and self-assembly of nanostructures. However, very few systematic studies of self-assembly using the grain coalescence, especially for three-dimensional (3D) nanostructures, exist at present. Here, we investigate the mechanism of plasma triggered grain coalescence to achieve the precise control of nanoscale phase and morphology of the grain coalescence induced by exothermic energy. Exothermic energy is generated through etching a silicon substrate via application of plasma. By tuning the plasma power and the flow rates of reactive gases, different etching rates and profiles can be achieved, resulting in various morphologies of grain coalescence. Balancing the isotropic/anisotropic substrate etching profile and the etching rate makes it possible to simultaneously release 2D nanostructures from the substrate and induce enough surface tension force, generated by grain coalescence, to form 3D nanostructures. Diverse morphologies of 3D nanostructures have been obtained by the grain coalescence, and a strategy to achieve self-assembly, resulting in desired 3D nanostructures, has been proposed and demonstrated.

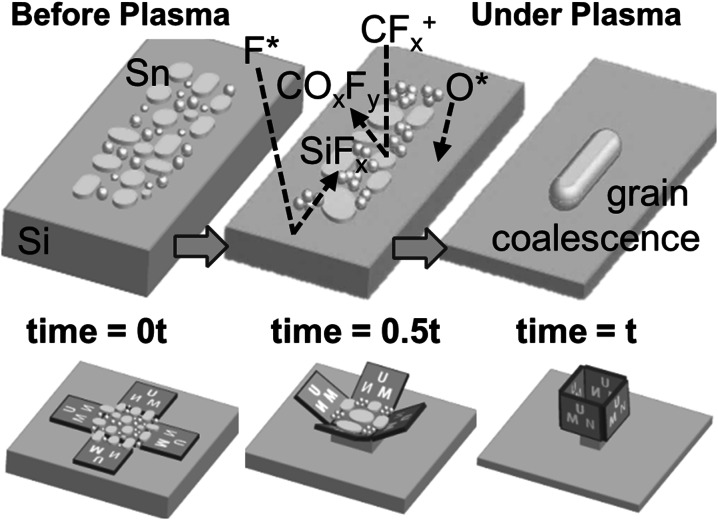

## Highlights

- Nanoscale grain coalescence induced during a Si etching process in a plasma etching system is characterized for self-assembly of 3D structures.
- During the etching process, balancing the isotropic/anisotropic substrate etching profile and the etching rate make it possible to simultaneously release 2D nanostructures from the substrate and induce enough surface tension force, generated by grain coalescence, to form 3D nanostructures.

## Introduction

Nanoscale metal grain coalescence in thin films after deposition is a process which accompanies sintering and densification of nanoparticles [1]. Phase and morphology changes resulting from grain coalescence induce advanced properties, which have been utilized in changing thin-film dielectric properties [2, 3], nanowelding [4–6], and self-assembly of 3D nanostructures [7–13]. In particular, the use of grain coalescence for the self-assembly of 3D nanostructures attracts great attention due to the ability of these structures to explore new physical and chemical phenomena for building next generation nanodevices.

In this assembly process, as a result of grain coalescence, a surface tension force is induced in metal thin films, when nanoscale grains liquefy. The surface tension force curves [7, 8, 10, 12, 13] or rotates [7, 9, 11–13] the underlying panels out of plane forming the 3D nanostructures. Since heat is the key factor that is responsible for melting the grains and triggering grain coalescence, a controllable heat source is required to achieve the desired grain coalescence performance. Plasma surface reaction, which enables controllable heat generation, is one of the approaches for triggering grain coalescence and controlling the coalescence performance [14–16]. Specifically, numerous plasma etching systems exhibit localized heat generation in the area of plasma/surface interaction [17–22]. The plasmochemical reactions in these plasma etching systems are exothermic and able to thermally stimulate a wide range of physical processes and chemical reactions [21, 22]. In addition, ion bombardment also happens in plasma etching systems, which contributes to heat generation by transferring kinetic energy [23–26]. As a result, the extreme heat generated on the surface of the reaction area is able to melt the grains and trigger grain coalescence in metal thin films [7–13]. The heat generation is determined by the reaction rate of the plasma etching, which can be controlled by plasma power and ratio of gas flow rates. Therefore, the performance of grain coalescence can be thermally controlled by tuning the reaction parameters. Although grain coalescence in tin (Sn) metal films was previously demonstrated in a reactive ion etching (RIE) system, which utilized the principle of plasma etching, and surface tension force generated by grain coalescence was used for assembly of both curved and polyhedron nanostructures [7–13], the effect of different conditions in the plasma etching system on grain coalescence has not been systematically studied. This brings great challenges in controlling the performance of grain coalescence, making the assembly of 3D nanostructures difficult to be reproduced on different structures.

In this work, we take an advantage of the controllable heat generation of plasma etching to achieve the desired grain coalescence for a self-assembly process. The grain coalescence in Sn thin films induced by RIE of silicon substrates with the gases tetrafluoromethane (CF_4_) and oxygen (O_2_) has been explored, and it is found that the power and ratio of gas flow rates (CF_4_/O_2_) in a RIE process show great effects on the morphologies of the grain coalescence as well as the self-assembled 3D structures. In addition, the effects of the different grain coalescence performances and Si substrate etching profiles on the self-assembly process have been studied. Finally, an approach to control the self-assembly of 3D nanostructures has been developed.

## Experimental Section

Three structures, Sn strips, 2D nets of cubic structure, and 2D ribbon, are used for studying the grain coalescence and their effects on self-assembly, respectively. The following is the fabrication process of these structures:

### Fabrication of Sn Strips

**Fig. 1:**
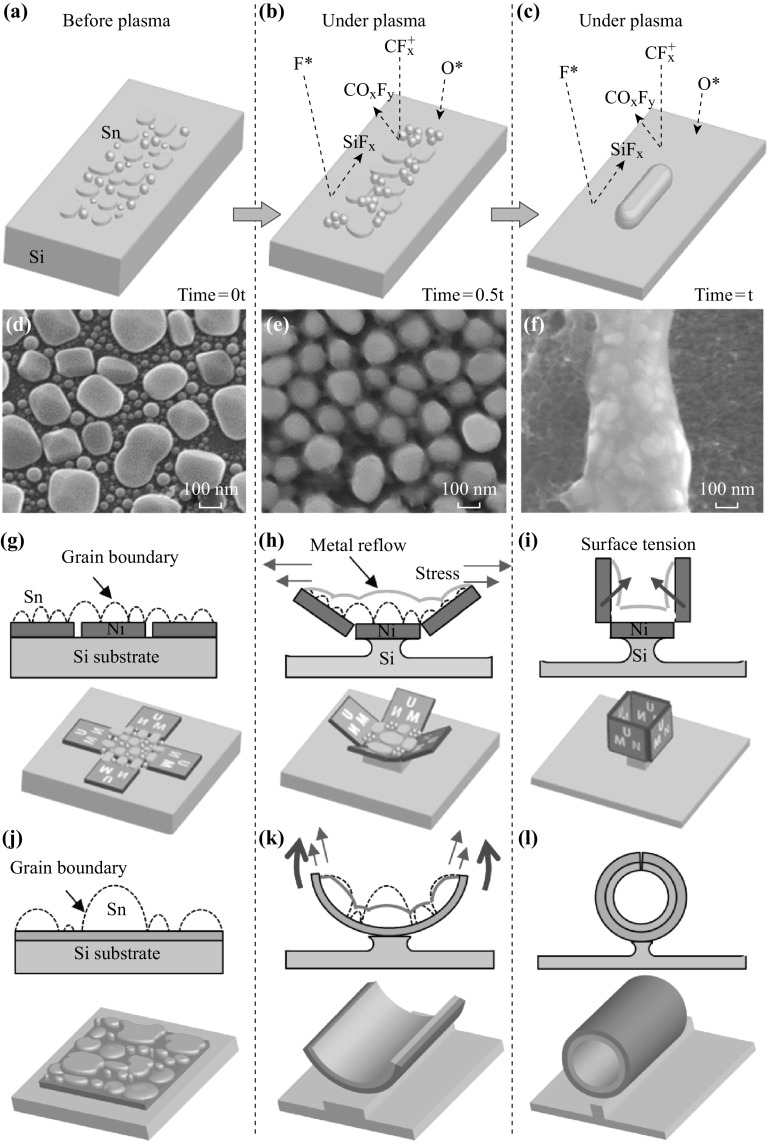
Conceptual schematics and SEM images showing the grain coalescence and self-assembly of 3D nanostructures triggered by reactive ion etching (RIE). **a–f** The grain coalescence process of Sn thin film during RIE process. **g–l** Illustrations showing the origin of the extrinsic stress and surface tension force observed within the Sn film that causes the **g–i** discontinuous frame to fold up or **j–l** the continuous film to curve up

To study the grain coalescence induced under plasma, 1-µm-wide strips were patterned by a lift-off process on a silicon (Si) wafer and then a 30-nm-thick Sn film was directly deposited on the Si substrate using an electron beam evaporator (Fig. 1a, d). The RIE of Si with CF_4_/O_2_ was used to generate heat energy and trigger grain coalescence in the sample placed on the chuck, which is a powered RF electrode. Various parameters, such as the plasma power and ratio of gas flow rates (CF_4_/O_2_), were changed to investigate and control the morphology of the grain coalescence. The status of grain coalescence (*γ*_g_) was quantified by calculating the exposed area of the Si substrate between the grains within the Sn strip divided by the original area of the Sn strip by using software (Polygonal Lasso Tool in Photoshop) analysis of scanning electron microscope (SEM) images (*γ*_g_ = exposed Si substrate after a RIE process/original area of Sn strip before a RIE process).

### Fabrication of 2D Nets for Self-Assembly of Cubic Structures

To utilize grain coalescence for triggering self-assembly of 3D nanostructures, 20-nm-thick nickel (Ni) 2D nets with five square nanoscale panels (with dimensions of ~500 × 500 nm^2^) were first defined, with “UMN” lettered patterns on the surrounding four panels, on a Si wafer by an electron beam lithography (EBL) process. On top of the panels, a 30-nm-thick Sn hinge was patterned. After the EBL process, reactive ion etching with CF_4_/O_2_ was used to induce Sn grain coalescence and realize the assembly of 3D nanostructures from the 2D nets.

### Fabrication of 2D Ribbon for Self-Assembly of Tube Structures

To demonstrate the mechanism can be applied to different structures, 2D ribbons (with dimensions of ~300 × 2000 nm^2^) were defined on a Si wafer by an electron beam lithography (EBL) process. After developing, 3-nm aluminum oxide (Al_2_O_3_) and 2.5-nm Sn were deposited to form the ribbon. After the lift-off process, reactive ion etching with CF_4_/O_2_ was used to induce Sn grain coalescence and realize the assembly of tube structures.

### Etch Rate Measurement

To measure the vertical and horizontal etch rate, a 10-nm-thick Ni pattern (4  × 4 μm^2^) is formed on the Si wafer. Reactive etching processes, with various powers and the ratio of gas flow rates, have been done on a group of the samples. After etching, Alpha Step 500 Profiler is used to measure the vertical etching rate by scanning across the boundary of the Si and Ni pattern. As the contrast is different for the etched and not etched area under the Ni pattern, scanning electron microscope (SEM, JEOL 6700) is used to measure the horizontal etch rate.

## Results and Discussion

In this study, 1-µm-wide Sn strips were fabricated to investigate the effect of plasma in a RIE system on grain coalescence and 2D nets of cubic and ribbon structures are used to explore the effect of grain coalescence and etching profile on self-assembly. The fabrication processes are described in the experimental section. The Sn film shows grainy morphology on the Si, nickel, and Al_2_O_3_ substrate because the surface tension of the Sn film exceeds that of the substrate [8] (Fig. 1a, d). To generate heat energy and trigger grain coalescence, the RIE of Si with CF_4_/O_2_ was used. In the plasma etching process, the chemical reaction between fluorine radicals and Si atoms produces the volatile products difluorosilicon (SiF_2_) and silicon tetrafluoride (SiF_4_) and is able to remove Si atoms from the surface [23, 27]. Additionally, as a feature of RIE, the ion bombardment (physical interaction) of active ion species like CF_x_^+^ also contributes to the etching (Fig. 1b, c) [28]. Both the chemical and physical reactions in the RIE process are exothermic and can generate localized extreme heat energy at the interface of the plasma and the Si substrate. As a result of the localized heat generated, the small Sn grains deposited on the Si substrate will start to melt and merge into large grains (Fig. 1b, e). With further etching of Si, the large grains will melt and coalesce with each other, forming one unit (Fig. 1c, f). The grain coalescence happens at the same time of etching process with the presence of plasma. As a consequence of this metal reflow, a surface tension force is induced in the Sn thin film. Once the grain coalesce is triggered in the Sn hinges of 2D structures, the surface tension force can lift up (Fig. 1g–i) or curve up (Fig. 1j–l) the underlying layers out of the plane (Fig. 1h, k), transforming them into 3D nanostructures (Fig. 1i, l).

**Fig. 2:**
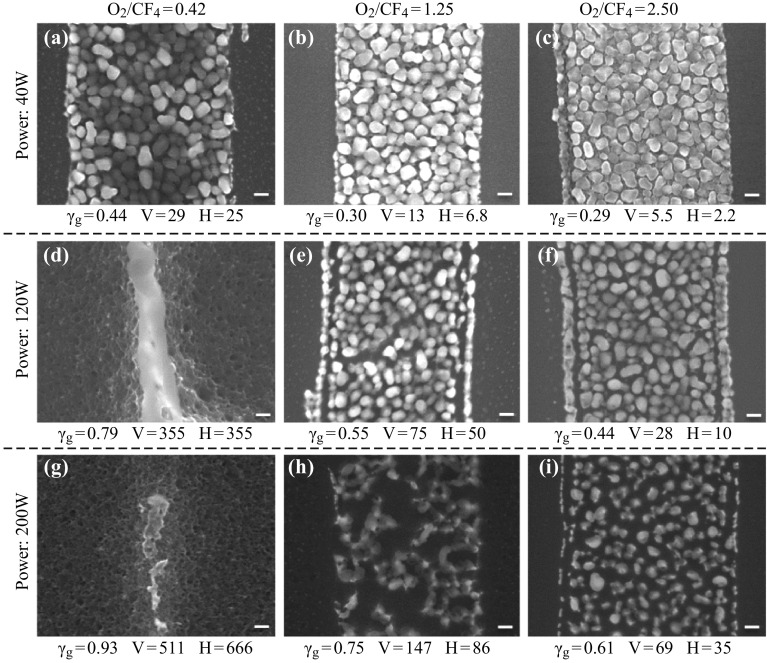
SEM images of the grain coalescence after a 4 min 30 s RIE process with varying power (40, 120, and 200 W) and ratio of O_2_ over CF_4_ (O_2_/CF_4_ = 0.42, 1.25, and 2.50). The pressure and CF_4_ flow rate are fixed to be 100 mTorr and 12 sccm, respectively. A series of oxygen flow rates of 5, 15, and 30 sccm was applied to change the ratio of O_2_ over CF_4_. The value of quantified grain coalescence, *vertical etch rate*, and *horizontal etch rate* of each sample was labeled as *γ*_g_ (a.u.), *V* (nm min^−1^), and *H* (nm min^−1^), respectively. The *scale bars* are 100 nm

**Fig. 3:**
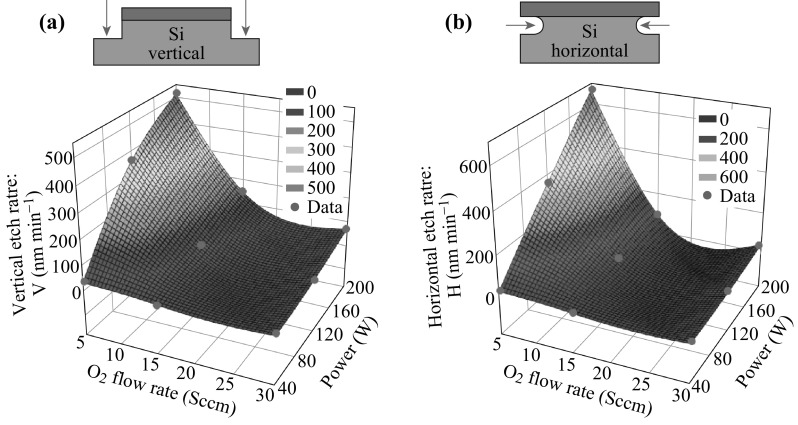
Experimental results of the variation of **a** *vertical etch* *rate *(*V*), and **b** *horizontal etch* *rate *(*H*) in the RIE process with varying power (40, 120, and 200 W) and varying ratio of O_2_ over CF_4_ (0.42, 1.25, and 2.50). The pressure and CF_4_ flow rate are fixed to be 100 mTorr and 12 sccm, respectively. Oxygen flow rates of 5, 15, and 30 sccm are utilized to control the ratio of oxygen over CF_4_

To understand and control the grain coalescence triggered by RIE, the effects of plasma power, ratio of gas flow rates, and etching rate of the Si substrate were systematically explored. In the experiment, the pressure and etching time were kept constant at 100 mTorr and 4 min 30 s, and a series of plasma powers of 40, 120, and 200 W were applied in a RIE system (STS Etcher 320). A fixed CF_4_ flow rate of 12 sccm combined with varying O_2_ flow rates of 5, 15, and 30 sccm was used in the RIE process, resulting in ratios (O_2_/CF_4_) of 0.42, 1.25, and 2.50. The various morphologies of grain coalescence caused by different combinations of power and ratio of gas flow rates were clearly observed (Fig. 2). As shown in Fig. 2, while a higher ratio of O_2_/CF_4_ shows an inhibition on the coalescence, an increased power contributes to greater grain coalescence. The observation was rationalized by noting the plasma power and ratio of flow rates are essential for the etch rate, thereby affecting heat generation, resulting in different morphologies of grain coalescence. On the one hand, plasma power of a RIE process influences the fluorine concentration [29]. At higher plasma power, more CF_4_ gas molecules can be dissociated due to the sufficient high electron energy [29]. More fluorine atoms in the gas molecules can be released by the dissociational collisions and lead to a higher fluorine concentration than at low plasma power, which enhances the chemical etch rate of the Si substrate [29]. On the other hand, the kinetic energy of the ions, such as CF_*x*_^+^, will also be enhanced due to high bias voltages (plasma power). As a result, the ion bombardment would be more significant and contribute to a higher physical etch rate. Associated with the enhanced etch rate due to both chemical and physical etching, more heat is generated on the substrate, which induces a more significant morphological change in the Sn grains meaning greater grain coalescence (Fig. 2). Moreover, additional O_2_ in the RIE chamber can initially enhance the etching due to the additional atomic fluorine created by the reaction between O_2_ and radicals like CF_3_ [30, 31]. Previous work has observed greater grain coalescence caused by increasing the O_2_ flow rate in this regime, where the percentage of O_2_ in the mixture gas is low [9]. When the percentage of O_2_ exceeds 5% in the gas mixture, which is the case discussed in this paper, a significant passivation film of SiF_x_O_y_ can be formed on the surface due to the high O_2_ flow rate [32]. The passivation layer cannot be removed by fluorine atoms, and it prevents the sputtering of Si by the CF_*x*_^+^ ion bombardment. Hence, the etch rate was greatly reduced (see Fig. 3). This low etch rate of Si resulted in less heat generation and no obvious grain coalescence. Therefore, the clear effect of the etch rate, as a function of gas flow rate and plasma power, on grain coalescence demonstrates that localized heat generated in plasma etching triggers the grain coalescence.

**Fig. 4:**
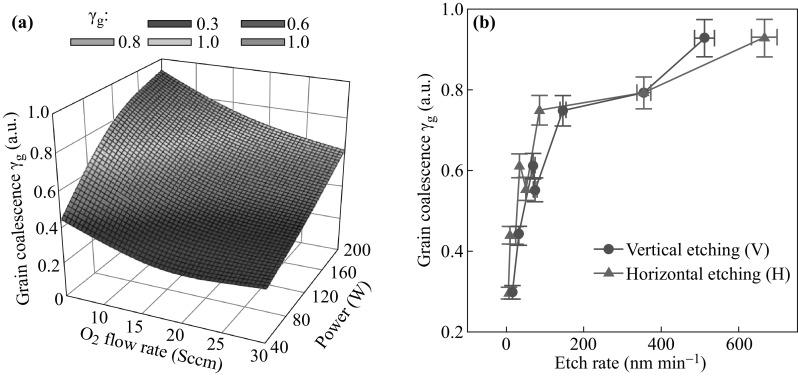
**a** Quantified grain coalescence in a RIE process with varying power (40, 120, and 200 W) and varying ratio of O_2_ over CF_4_ (0.42, 1.25, and 2.50). The pressure and CF_4_ flow rate are fixed to be 100 mTorr and 12 sccm, respectively. Oxygen flow rates of 5, 15, and 30 sccm are utilized to control the ratio of oxygen over CF_4_. **b** The relationship between the grain coalescence and both the *vertical *and the *horizontal etch rates*

During the grain coalescence, the small grains merge into each other to form large grains to reduce surface area for minimizing surface energy. Therefore, the surface area of the grains can be used as a parameter to evaluate the grain coalescence. To quantitatively study the effect of the plasma parameters (i.e., gas flow rate, plasma power, and etch rate) on the grain coalescence, the status of grain coalescence (*γ*_*g*_) was quantified by calculating the exposed area of Si substrate between the grains, within the Sn strip, divided by the original area of the Sn strip by using software (Polygonal Lasso Tool in Photoshop). As a 1-μm-wide Sn strip is defined on the Si substrate, even though the Sn grain flows in a few hundred nanometers after melting, Sn still stays within the area that was captured by SEM images zoomed out for the characterization of *γ*_g_. In addition, the data are collected based on average value of grains, which can minimize the error. Higher values of *γ*_g_ indicate more significant grain coalescence (Figs. 2, 4a). The data shown in Fig. 4 were generated from Fig. 2. It is clear *γ*_g_ was more significant with lower oxygen flow rate and higher plasma power (Fig. 4a), which was consistent with both the changing trend of horizontal and vertical etching rates according to the oxygen flow rate and plasma power shown in Fig. 3. The oxygen flow rate affects the etching rate, which affects grain coalescence. With high flow rates of O_2_, a passivation layer of SiF_*x*_O_*y*_ forms on the surface of the Si substrate, resulting in a low Si etch rate and less heat generation. Therefore, morphology of weak grain coalescence (low *γ*_g_) was observed for trials with higher oxygen flow rates (Fig. 4a). Increasing the plasma power leads to an increase in the atomic fluorine concentration, enhancing the chemical reaction for etching Si. Additionally, the high plasma power intensifies ion bombardment, increasing thermal energy transferred to the Sn grains [23, 24]. Since both the chemical and physical reactions are exothermic, a high etching rate contributes to greater heat generation, resulting in more significant grain coalescence.

To further investigate the mechanism of grain coalescence, the direct relationship between grain coalescence and both the vertical and the horizontal etching rate are plotted (Fig. 4b). As shown in Fig. 4, the grain coalescence shows an overall increasing behavior with both increasing vertical and horizontal etching rate until the etch rate of 100 nm min^−1^ is reached. However, as the etch rate increases further (>100 nm min^−1^), the amount of grain coalescence saturates. This could be caused by the limited volume of the Sn grains. Since the volumes of the Sn grains are in nanoscale, the heat generated on the Si substrate, within the high etch rate region, can easily reach the required level for triggering significant grain coalescence. Once significant grain coalescence has been triggered, further increase in the heat generation cannot significantly speed up the process due to the limited volume of the Sn grains, thereby causing the saturation region (Fig. 4b). The results demonstrate grain coalescence can be easily controlled by adjusting the etching rate of the Si substrate; thereby indicating self-assembly of 3D nanostructures utilizing grain coalescence can be precisely controlled.

**Fig. 5:**
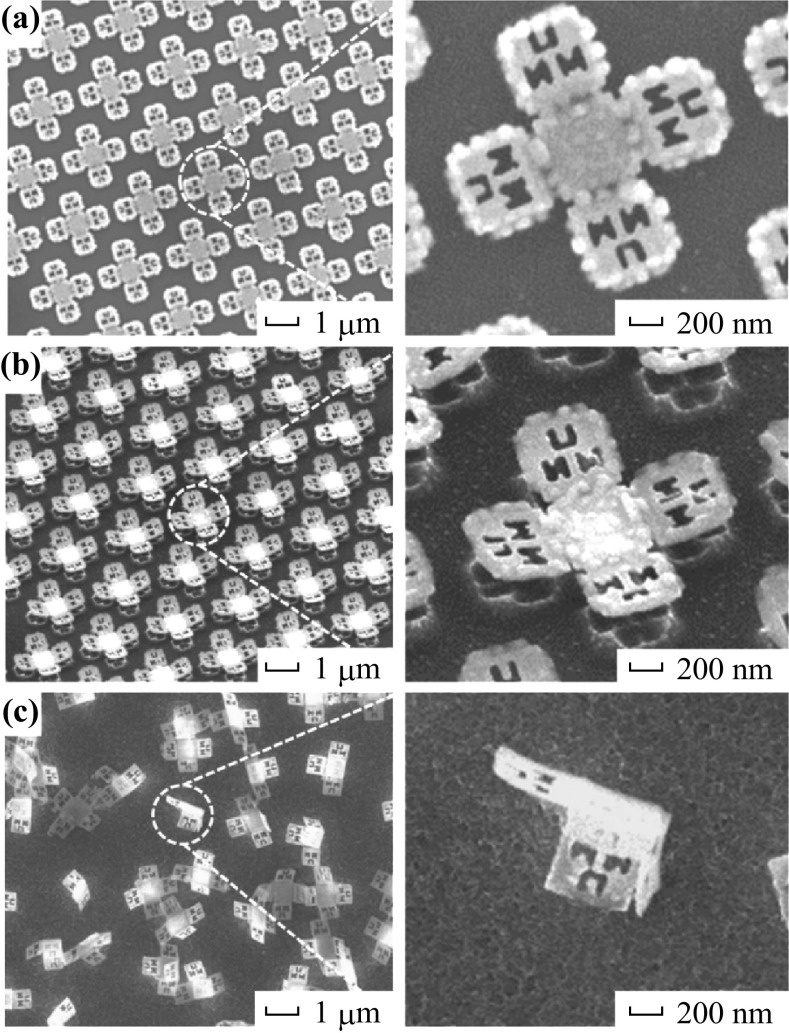
SEM images of self-assembly of 3D nanostructures with different oxygen flow rates. A pressure of 100 mTorr, power of 120 W, CF_4_ flow rate of 12 sccm, and an etching time of 4 min 30 s were used. **a** At an oxygen flow rate of 30 sccm, the structures were not able to be folded due to the low etch rate. **b** At an oxygen flow rate of 15 sccm, the nanostructures are uniformly folded with an angle of 45° based on the stress generated in the moderate grain coalescence. **c** At oxygen flow rate of 5 sccm, considerable heat generation and grain coalescence do not contribute to fully folded structures. Only parts of the panels get folded more than 90°

To apply the mechanism observed in Fig. 4 and realize 3D nanostructures, 2D nets of cubic structures were fabricated as described in the experimental section. After fabrication of 2D nets, RIE was used to induce Sn grain coalescence. Associated with the grain coalescence, a surface tension force is induced in the hinge, transforming the 2D nets into 3D nanostructures (Fig. 1). To investigate the effect of grain coalescence on nanoscale self-assembly, the pressure and etching time of the RIE process for self-assembly were fixed at 100 mTorr and 4 min 30 s, respectively, which are the same values as used in Fig. 2. The plasma power of 120 W and varied ratio of gas flow rates (O_2_/CF_4_) of 0.42, 1.25, and 2.50 were selected because these conditions showed dramatically different grain coalescence (*γ*_g_ = 0.791, 0.552, and 0.437, respectively) (Fig. 2d–f). With the gas flow ratio of 2.50, no significant grain coalescence (*γ*_g_ = 0.437) was observed. This indicates the surface tension forces generated in the hinges are not sufficient to fold the panels and transform the 2D nets into 3D nanostructures even though the panels were completely released from the substrate (Fig. 5a). With a lower gas flow rate ratio (1.25), the grain coalescence with some portion of large grains was triggered in the Sn hinges, which induces surface tension forces in the hinges and transforms the 2D nets into uniform 3D nanostructures with a folding angle of 45° (Fig. 5b) (time for applying RIE was ~4 min 30 s). In order to trigger considerable grain coalescence and fully fold the 2D nets into 3D nanocubes with an angle of 90°, a lower gas flow ratio of 0.42 was applied (Fig. 2d). However, only parts of the panels were folded to an angle of 90° and no successfully folded 3D nanostructures were observed for this recipe (Fig. 5c). The reason is because it is insufficient to only control the surface tension force for achieving the uniformly assembled (homogeneous) 3D nanostructures. To achieve completely folded, homogeneous, 3D nanostructures, the grain coalescence and release of the panels from the substrate should be balanced. Sufficient grain coalescence should be triggered while the surrounding panels are released; however, the central panel should still be connected to the substrate until the structure is completely folded. If the central panel is released from the substrate, no stable connection exists between the Si substrate and the nanostructures, which makes it difficult to uniformly transfer the localized heat to the structures; hence, no uniform 3D structure can be achieved. Therefore, the release of the panels, which is determined by the etching profiles, should also be precisely controlled.

**Fig. 6:**
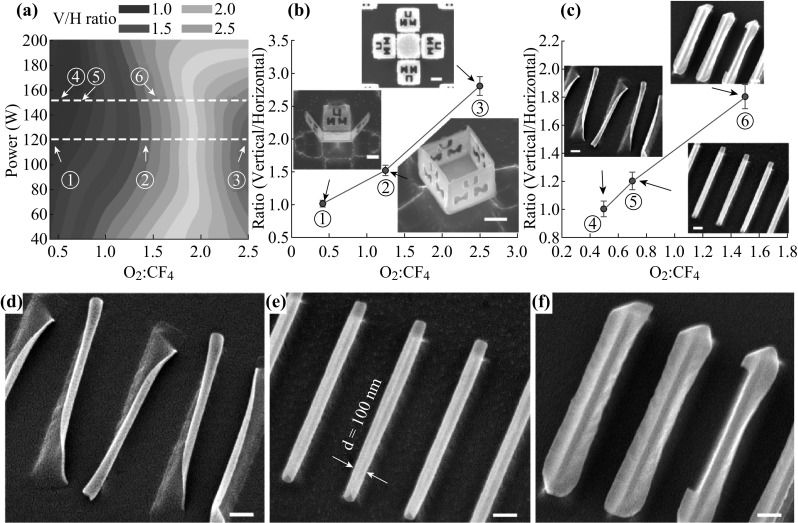
Experimental results and SEM images. **a** The ratio of *vertical etch* *rate* over *horizontal etch* *rate* in a RIE system with fixed pressure of 100 mTorr and various power and gas flow rate ratio. The effect of etch rate ratio on self-assembly of **b** cubic structures with a fixed power of 120 W and **c** tube structures with a fixed power of 150 W. The six data points in **b** and **c** correspond to the labeled points ①–⑥ in (**a**). **d–f** The zoom in image of the insets of **c** with schematic illustration. A thin layer of Au (~3 nm) is coated on top of tube structures for imaging. The *scale bar* is 200 nm

The RIE process is responsible for not only triggering grain coalescence but also releasing the surrounding panels from the substrate. During the self-assembly process, both vertical and horizontal etching occur in the RIE system. However, if the horizontal etching much more actively occurs, the structures will be released before sufficient grain coalescence is triggered, causing the structures to not be fully folded. On the other hand, if the horizontal etching rate is much lower than the vertical etching rate, the surrounding panels will not be released during the Sn grain coalescence, resulting in unfolded structures. Therefore, for self-assembly of 3D nanostructures, the ratio of vertical etch rate over horizontal etch rate needs to be tuned to generate enough heat energy while only the surrounding panels are completely released. For controlling the etch rate ratio (vertical/horizontal), the ratio of O_2_ flow rate over CF_4_ is the dominant factor [23]. At low gas flow rate ratios (O_2_/CF_4_), chemical reactions between fluorine and silicon atoms dominate the etching process. As the chemical reaction has no directionality, the etching profile is isotropic, which means the etch rate ratio (vertical/horizontal) is around 1 (Fig. 6a). With an increasing gas flow rate ratio, a passivation layer of SiF_*x*_O_*y*_, induced by high oxygen flow rate, forms on the surface of the silicon substrate. The passivation layer can only be removed by ion bombardment rather than the chemical reaction. As the ion bombardments are in the vertical direction, the vertical etch rate (*V*) becomes more significant compared to horizontal etch rate (*H*) (Fig. 6a). Therefore, increasing gas flow rate ratio (O_2_/CF_4_) could monotonically increase the ratio (*V*/*H*) of vertical etch rate over horizontal etch rate (Fig. 6a).

The understanding of both the grain coalescence and etching profile offers a strategy to analyze the self-assembly process and achieve fully folded 3D structures. At a low oxygen flow rate of 5 sccm (O_2_/CF_4_ = 0.42), the ratio of vertical etch rate over horizontal etch rate (*V*/*H*) is low. At the point in time when the nanostructure is fully released from the substrate, a relatively low amount of Si is vertically etched, which results in insufficient heat generation, leading to insignificant grain coalescence. Hence, there is not enough surface tension force generated in the Sn hinge films to fold all surrounding panels up to 90° until right after the central panel has already been released from the Si substrate (Fig. 6b, the left inset SEM image pointing ①, the RIE conditions highlighted with a dash line in Fig. 6a correspond to the RIE conditions and results shown with a solid line in Fig. 6b). Once the structure is released from the substrate, there is no stable connection between the nanostructures and the substrate, making it difficult to uniformly transfer heat to the nanostructures. Even though significant heat has been generated by further application of RIE after the central panel is released, there is still no sufficient grain coalescence in some of the Sn hinges due to the difficulty of heat transfer. As a result, no uniformly folded 3D nanostructures can be achieved (Fig. 5c). In addition, significantly increasing the etch rate ratio to 2.80 by applying a high oxygen flow rate of 30 sccm (O_2_/CF_4_ = 2.50), which relatively increases vertical etching and decreases horizontal etching, is also not able to achieve successful self-assembly. The low horizontal etch rate contributes to a long etching time to release the surrounding panels. During this process, a large amount of Si can be etched because of the long etching time and relatively high vertical etching rate, making the Sn hinge completely melt and disperse away from the panels before the surrounding panels are released. Since the Sn hinges on and between the panels were already removed prior to the release of the outer panels from the substrate, the 2D nets can no longer be folded (Fig. 6b, the right inset SEM image pointing ③). Therefore, by considering both the grain coalescence and etching profile, an oxygen flow rate of 15 sccm (O_2_/CF_4_ = 1.25), which leads to an etch rate ratio of 1.50, was used. With this recipe, the assembly of completely folded 3D structures is successfully performed because sufficient surface tension force is induced in the thin films in proper timing with the release of the surrounding panels, while the central panel is still connected (Fig. 6b, the middle inset SEM image pointing ②). Therefore, a recipe with a pressure of 100 mTorr, power of 120 W, CF_4_ flow rate of 12 sccm, O_2_ flow rate of 15 sccm, and RIE time of 5 min 30 s shows the ability to assemble the 3D polyhedral nanostructures. As the structures with different size and shape require different heat generation and structure release process, this recipe is not applicable to all the self-assembly process. However, the mechanism used for achieving this recipe can be used as guidance for self-assembly, which will greatly reduce the time for optimizing the recipe. For new structures, different power should be first tried to achieve initial folding or curving performance even though it is not completely or uniformly self-assembled. Then, the gas flow rate ratio should be tuned to optimize the process for successful self-assembly.

This mechanism can be applied to self-assembly of polyhedral structures with discontinuous thick films as well as curved self-assembly structures such as curvature with continuous thin films under Sn grains (Fig. 1j–l). As shown in Fig. 6a–c, the strategy mentioned in this paper can be directly applied to self-assembly of tube structures. After figuring out the power of 150 W, the gas flow rate ratio was optimized to achieve the successful self-assembly process. The zoom in images of the insets of Fig. 6c are shown in Fig. 6d–f, corresponding to ④, ⑤, and ⑥. At the O_2_/CF_4_ ratio of 0.5, the vertical to horizontal etching rate ratio is 1, which cannot accumulate enough energy to self-assembly of the tube before releasing it form the substrate, resulting in ununiformed partially folded tube structure (Fig. 6c, d). By slight increasing the O_2_ to CF_4_ gas flow rate ratio, the vertical to horizontal etching rate ratio can be increased to 1.2, which can generate more energy for folding the structures before releasing, resulting in totally folded tube structure (Fig. 6c, e). Similar to the trend of cubic structures, further increase in the O_2_ to CF_4_ flow rate ratio will lead to higher vertical to horizontal etch rate ratio (1.8), which make the Sn melted and flowed away from surface before the structure can be folded, leading to the failure of self-assembly (Fig. 6c, f).

## Conclusion

We have characterized nanoscale grain coalescence induced by heat generated by a Si etching process in a plasma etching system. Plasma power and gas flow rate ratio in the RIE process have been shown to have effects on the etching rate, which affects heat generation, impacting grain coalescence. This study highlights how the performance of grain coalescence can be controlled by tuning the power and gas flow rate ratio between O_2_ and CF_4_. In addition, substrate etching rates and etching profiles have been demonstrated to be the dominant factors affecting the self-assembly of 3D nanostructures because they are responsible for inducing enough surface tension force and releasing the structures from the substrate, respectively. Also, the strategy has been demonstrated to be able to be used for various structures rather than only polyhedral structures. This study can serve as a guideline for self-assembly of 3D nanostructures utilizing nanoscale grain coalescence.
